# BYSL contributes to tumor growth by cooperating with the mTORC2 complex in gliomas

**DOI:** 10.20892/j.issn.2095-3941.2020.0096

**Published:** 2021-02-15

**Authors:** Shangfeng Gao, Zhuang Sha, Junbo Zhou, Yihao Wu, Yunnong Song, Cheng Li, Xuejiao Liu, Tong Zhang, Rutong Yu

**Affiliations:** 1 Institute of Nervous System Diseases, Xuzhou Medical University; Department of Neurosurgery, The Affiliated Hospital of Xuzhou Medical University, Xuzhou 221002, China

**Keywords:** Bystin, glioblastoma, proliferation, apoptosis, AKT

## Abstract

**Objective:**

*BYSL*, which encodes the Bystin protein in humans, is upregulated in reactive astrocytes following brain damage and/or inflammation. We aimed to determine the role and mechanism of BYSL in glioma cell growth and survival.

**Methods:**

BYSL expression in glioma tissues was measured by quantitative real-time PCR, Western blot, and immunohistochemistry. *In vitro* assays were performed to assess the role of BYSL in cell proliferation and apoptosis. Protein interactions and co-localization were determined by co-immunoprecipitation and double immunofluorescence. The expression and activity of the AKT/mTOR signaling molecules were determined by Western blot analysis, and the role of BYSL in glioma growth was confirmed in an orthotopic xenograft model.

**Results:**

The BYSL mRNA and protein levels were elevated in glioma tissues. Silencing BYSL inhibited glioma cell proliferation, impeded cell cycle progression, and induced apoptosis, whereas overexpressing BYSL protein led to the opposite effects. We identified a complex consisting of BYSL, RIOK2, and mTOR, and observed co-localization and positive correlations between BYSL and RIOK2 in glioma cells and tissues. Overexpressing BYSL or RIOK2 increased the expression and activity of AKT/mTOR signaling molecules, whereas downregulation of BYSL or RIOK2 decreased the activity of AKT/mTOR signaling molecules. Silencing BYSL or RIOK2 decreased the growth of the tumors and prolonged the lifespan of the animals in an orthotopic xenograft model.

**Conclusions:**

High expression of BYSL in gliomas promoted tumor cell growth and survival both *in vitro* and *in vivo.* These effects could be attributed to the association of BYSL with RIOK2 and mTOR, and the subsequent activation of AKT signaling.

## Introduction

Glioma is the most common and aggressive primary brain tumor. Although significant advances have been made in surgery and adjuvant therapy, patients with malignant glioma have experienced little change in survival time^1^. The current standard of care for patients with glioblastoma (GBM) is only palliation, which provides a median survival of approximately 12–15 months^2^. Investigating novel therapeutic targets to combat tumor growth and progression is therefore critical for the treatment of this currently incurable cancer.

*BYSL*, which encodes the Bystin protein in humans, is a gene that is evolutionarily conserved from yeast to humans^3^. The yeast *BYSL* homolog, *ENP1*, is localized in the nucleus and is essential for yeast vegetative growth and ribosome biosynthesis^3,4^. The Drosophila *BYSL* homolog, *bys*, is also localized to the nucleus, and its expression profile is similar to that of many Myc target genes^5^. These findings indicate a potential role of BYSL in cell growth.

It has been found that BYSL expression is elevated in lung cancer, prostate cancer, liver cancer, and ovarian cancer^6ߝ8^. *In vitro* and *in vivo* experiments have shown that BYSL promotes hepatocellular carcinoma (HCC) cell survival and tumorigenesis^6^. In addition, an increase in the transcriptional level of BYSL predicts a shorter survival time in breast cancer patients^9^. These studies suggest that BYSL may play an oncogenic role in cancer progression.

BYSL is upregulated in reactive astrocytes activated by brain damage and inflammatory mediators, and it has been considered to be a more sensitive marker of astrocyte proliferation than GFAP^10,11^. Thus, we hypothesized that BYSL may contribute to human glioma growth. In this study, we first investigated changes in the expression of BYSL in glioma tissues and analyzed the association of BYSL levels with patient overall survival using public datasets and our cohort. We then identified the role of BYSL in glioma cell proliferation and apoptosis using small interfering RNA (siRNA) or lentivirus-mediated overexpression of BYSL. Furthermore, we demonstrated that BYSL formed a complex with RIOK2 and mTOR, and that both BYSL and RIOK2 positively regulated AKT/mTOR signaling. Finally, intracranial xenograft experiments confirmed the oncogenic roles of BYSL and RIOK2 in glioma growth.

## Material and methods

### Patients and samples

All glioma tissue specimens (obtained during surgical resection) and nontumor brain tissue specimens (obtained from patients undergoing surgery for internal decompression after cerebral trauma) were collected from the Affiliated Hospital of Xuzhou Medical University. All patients were naïve to immunotherapy, radiation, and chemotherapy. For quantitative real-time PCR (qRT-PCR) and Western blot analysis, fresh samples were stored at −135 °C immediately after surgical removal. For immunohistochemical analysis, the specimens were fixed in 10% buffered formalin and embedded in paraffin for sectioning. The clinicopathological information of all patients is presented in **Supplementary Table S1**. All glioma specimens had a confirmed pathological diagnosis and were classified according to the criteria of the World Health Organization (WHO).

### Cell lines and cell culture

HEK293T cells and the human glioma cell lines, U251 and U87, were purchased from the Shanghai Cell Bank, Type Culture Collection Committee, Chinese Academy of Sciences (Shanghai, China). The identities of the U251 and U87 cell lines were confirmed by a DNA profiling test. The cells were grown in Dulbecco’s Minimal Eagle’s Medium (HEK293T and U251) or Minimal Essential Medium (U87) supplemented with 10% fetal bovine serum (Gibco, Grand Island, NY, USA). The primary glioma cell lines of two GBM cases were cultured using the enzyme digestion method as described in our previous reports^12,13^. All cell lines were cultured in a cell incubator with a 5% CO_2_ atmosphere under saturated humidity at 37 °C.

### Antibodies and plasmids

A rabbit anti-BYSL polyclonal antibody from Sigma-Aldrich (St. Louis, MO, USA) was used for Western blot (1:500) and immunohistochemistry (1:200) experiments. A rabbit anti-RIOK2 polyclonal antibody (1:50; Abnova, Taibei City, Taiwan) and a mouse anti-RIOK2 polyclonal antibody (1:400; Sigma-Aldrich) was used for Western blot and immunofluorescence, respectively. Magnetic FLAG beads (Sigma-Aldrich), Protein A/G PLUS-Agarose, and normal rabbit IgG (Santa Cruz Biotechnology, Santa Cruz, CA, USA) were used in the immunoprecipitation assays. Antibodies against β-actin (1:1,500; Santa Cruz Biotechnology), FLAG (1:1,000; Sigma-Aldrich), Myc, and the signaling molecules (1:1,000; Cell Signaling Technology, Danvers, MA, USA) were used for Western blot analysis. The Myc-tagged RIOK2-overexpressing plasmid was obtained from the Chinese Science Academy (Beijing, China)^14^, and the FLAG-tagged BYSL-overexpressing plasmid was purchased from Viogene Biosciences (Jinan, China).

### Public database analysis

The GSE16011 dataset was downloaded from the R2: microarray analysis and visualization platform (http://hgserver1.amc.nl), and the figure showing the differential expression was generated by Prism 8 (GraphPad, San Diego, CA, USA). Differences in BYSL expression between the low grade glioma (LGG) and GBM patients and their respective normal controls were determined online using the GEPIA web server^15^. The prognosis analysis of The Cancer Genome Atlas (TCGA) dataset, including both LGG and GBM, was performed online with the GEPIA web server^15^. The prognosis analysis of the mRNAseq_693 dataset, including 401 cases of all WHO grade gliomas, was performed using the CGGA website (http://www.cgga.org.cn). Correlation analyses of the LGG, GBM, and GSE16011 datasets were performed online. All figures and the related parameters were downloaded from the official websites.

### RNA extraction and qRT-PCR

Total RNA extraction and reverse transcription were performed as described in our recent report^16^. The target genes were amplified in a final volume of 20 μL with SYBR Green PCR Maser mix (Takara, Beijing, China) and a mixture of forward and reverse primers as follows: BYSL (forward: 5′-CTGGTTCAAAGGGATCCTGA-3′, reverse: 5′-AGTCGCAGGAAGATGCTGTT-3′); RIOK2 (forward: 5′-ACATGAGCCGAGATGACTTC-3′, reverse: 5′-AACCGATAGCCCTGGACA-3′); and β-actin (forward: 5′-CCAACCGCGAGAAGATGA-3′, reverse: 5′-CCAGAGGCGTACAGGGATAG-3′). The same PCR temperature cycles and real-time PCR system were used as previously described^16^. The data were acquired and processed automatically using the Applied Biosystems 7500 (Applied Biosystems, Foster City, CA, USA). The expressions of the target genes were normalized to that of β-actin, and the relative absolute expressions of the target genes were calculated according to our previous statistical method^17^.

### Protein extraction and Western blot

Total protein was extracted from the tissues or cultured cells, and the protein concentration was determined using a BCA Protein Assay Kit (Beyotime, Haimen, China). Western blot analysis was performed according to a previously reported protocol^16^. Primary antibodies were added at the dilution ratios described in the “Antibodies and plasmids” section. Band densities were quantified using ImageJ software (National Institutes of Health, Bethesda, MD, USA). Relative protein levels were determined by normalizing the densitometry value of the proteins of interest to that of β-actin or GAPDH.

### Immunohistochemistry

Immunohistochemistry was performed as described in our recent report^18^, with slight modifications. Briefly, antigen retrieval was conducted in sections in citrate buffer (pH 6.0) using microwave radiation, followed by incubation with 5% milk in Tris-buffered saline (pH 7.6) at room temperature for 2 h to reduce nonspecific staining. The sections were incubated with the BYSL polyclonal antibody for 2 h at room temperature followed by overnight incubation at 4 °C. All sections were then processed using an ABC kit (Vector Laboratories, Burlingame, CA, USA) according to the manufacturer’s protocol. Finally, the sections were incubated for 10 min in a substrate solution containing 0.01% hydrogen peroxide. The sections were counterstained with hematoxylin (KeyGEN BioTECH, Jiangsu, China). All images were captured using a DM2500 microscope (Leica, Wetzlar, Germany).

### Cell counting

The quantification of BYSL-immunoreactive staining was performed according to our previously reported method^16,19^. Briefly, the white matter of the nontumor tissues or the tumor centers of the glioma tissues were identified using low magnification (10×) based on the hematoxylin-stained nucleus. For each subject, three high magnification (40×) images were randomly selected and captured within the areas of interest. The total number of hematoxylin positive cells and the number of BYSL positive cells were counted by an investigator who was blinded to the subject identities. To prevent double counting, only positively stained cell profiles containing a nucleus were counted. The percentage of BYSL-IR cells was calculated by dividing the number of BYSL-IR cells by the total number of cells in each field, and the average percentage of 3 fields was used for statistical analysis.

### Transfection

For siRNA transfection, we used previously validated siRNA to downregulate the *BYSL* transcript^6^. The siRNAs were synthesized by Biomics Biotech (Nantong, China). Once the cells reached 70%–90% confluence in 6-well plates, BYSL siRNA (siBYSL) or negative control siRNA (siNC) was transfected using Lipofectamine 2000 (Invitrogen, Carlsbad, CA, USA) according to the manufacturer’s instructions.

For plasmid transfection, when the cells reached a confluence of 40%–50% in 6 cm dishes, 1 μg of plasmid was transfected with PolyJet (SignaGen, Gaithersburg, MD, USA) according to the protocol provided by the manufacturer. Six hours later, the medium was replaced with 2 mL fresh medium, and the cells were further incubated for 48 h before protein extraction.

### Lentivirus construction, production, and infection

Human *BYSL* (Accession number: NM_004053) was inserted into the pCDH-GFP-puro vector at the Nhe I and Bgl II sites, and human *RIOK2* (Accession number: NM_018343) was inserted into the same vector at the Nhe I and BamH I sites. The short-hairpin RNA (shRNA) oligomers were subcloned into the pLV-puro plasmid at the BamH I and EcoR 1 sites. The sense target sequences were as follows: Scramble (5′-GCGTCGTCCAACATTATCAT-3′), shRIOK2-2 (5′-GTCCAGGGCTATCGGTTGA-3′), and shRIOK2-4, (5′-GAACGGAACTGTCTAGAAG-3′). These lentiviruses were produced in HEK293T cells and were used to infect glioma cells according to a previously reported protocol^20^. Forty-eight hours after infection, the virus-infected cells were cultured in medium containing 2.5 μg/mL puromycin for selection. The surviving cells were used in subsequent experiments.

### Cell growth assay

Cell viability was measured with a Cell Counting Kit-8 (CCK-8; Dojindo Molecular Technologies, Kumamoto, Japan). A single cell suspension (5 × 10^3^ cells/well) was seeded in a 96-well plate and cultured for 6, 24, 48, and 72 h. The CCK-8 reagent was added to each well and incubated for another 1.5 h at 37 °C. Then, the absorbance (value) was measured at 450 nm in an automated plate reader (BioTek, Winooski, VT, USA). The cell viabilities at individual time points were normalized to those at 6 h.

### The 5-ethynyl-20-deoxyuridine (EdU) incorporation assay

The cells were seeded in 96-well plates at 5 × 10^3^ per well, and 24 h later, an EdU assay was performed using a commercial kit (RiboBio, Guangzhou, China) according to the manufacturer’s instructions. The cell nuclei were stained with 100 μL of Hoechst 33342 (5 μg/mL). The images were acquired using an IX-71 inverted microscope (Olympus, Tokyo, Japan). The percentage of EdU-positive cells was calculated by dividing the number of EdU-positive cells by the number of Hoechst-stained cells.

### Flow cytometry

The cell cycle was assessed by flow cytometry using a commercial kit (NewMed Cytomics, Suzhou, China) according to the manufacturer’s protocol. The cells were trypsinized, resuspended into single cell suspensions, and collected by centrifugation at 1,500 rpm. Reagents A, B, and C from the kit were successively added to the cells. The cell suspension was filtered and immediately analyzed by flow cytometry (BD Biosciences, Franklin Lakes, NJ, USA).

### Flow cytometry apoptosis assay

The cells were harvested and washed twice with ice-cold phosphate-buffered saline and then resuspended in 1× binding buffer [0.01 M HEPES/NaOH (pH 7.4), 0.14 M NaCl, 2.5 mM CaCl_2_] at a concentration of 10^6^ cells/mL. A total of 100 μL of the solution was transferred to a 5 mL cell culture tube and then treated with a fluorescein isothiocyanate-conjugated annexin V apoptosis detection kit I (BD Biosciences) according to the manufacturer’s protocol. The cells were analyzed using a flow cytometry system (BD Biosciences), and a total of 10,000 cells per sample were analyzed to determine the percentage of apoptotic cells.

### Co-immunoprecipitation

After transient transfection with the indicated plasmids, the adherent cells were lysed with ice-cold cell lysis buffer (Beyotime). The lysate was collected and then centrifuged at 10,000 × *g* for 10 min to collect the supernatant. One mg of total protein was precleared with Protein A/G PLUS-Agarose at 4 °C for 1 h, and then it was subjected to immunoprecipitation (IP) with magnetic FLAG beads or the appropriate IgG at 4 °C overnight. Protein A/G PLUS-Agarose beads were then added and incubated for 2 h. The immunoprecipitated proteins were eluted with equal amounts of 2× protein-loading buffer and subjected to Western blot analysis.

### Double labeling immunofluorescence

Immunofluorescence was performed as previously described^21,22^. Briefly, cells were fixed with 4% formaldehyde, permeabilized with 0.1% Triton X-100, and incubated with 3% (w/v) bovine serum albumin to block nonspecific sites. Then, the following primary antibodies were simultaneously added: (i) mouse anti-RIOK2 (1:400) and (ii) rabbit anti-BYSL (1:200). RIOK2 was visualized in red by anti-mouse Alexa594 (1:200; Invitrogen). BYSL was detected in green by anti-rabbit Alexa488 (1:400, Invitrogen). The cell nuclei were stained with 4,6-diamidino-2-phenylindole (DAPI; 1:1,000; Sigma-Aldrich) for 10 min. The sections were immediately cover slip mounted with Permount (Haoran Biological Technology, Shanghai, China) and observed using a LSM880 AiryScan confocal microscope (Zeiss, Oberkochen, Germany).

### Intracranial xenograft model and histopathology

An intracranial xenograft model was established to study the effects of the downregulation of RIOK2 or BYSL on the growth of glioma *in vivo*. Male athymic BALB/c nude mice aged 5–6 w and weighing ˜20 g were obtained from the Experimental Animal Center of Xuzhou Medical University. After the mice were anesthetized, a small burr hole (2 mm in diameter, 1 mm lateral to the midline, and 2 mm posterior to the coronal suture) was drilled on the right side of the cranium. Then, 5 × 10^5^ U87 cells were intracranially inoculated into the right striatum of the nude mice as described in our previous study^23^. Mice were sacrificed at the onset of cachexia or at 4 weeks following the inoculation of the glioma cells. The whole brains were isolated, fixed, and dehydrated sequentially in 20% and 30% sucrose at 4 °C until they sank. The freshly frozen brains were continuously sectioned at a thickness of 12 μm using a cryostat microtome (Leica), and the section with the largest transactional tumor area was subjected to hematoxylin and eosin staining. The tumor volume was calculated according to the formula: V = 1/2 ab^2^, where a and b indicate the longest and shortest diameters, respectively.

### Ethical approval

Written informed consent was obtained from each subject or legal guardian and signed by patients and legal guardians prior to participation in the study. The research was conducted in accordance with the Declaration of Helsinki (as revised in 2013), and the experimental protocol was approved by the Ethics Committee of Xuzhou Medical University (Approval No. EA20171225).

The mice were housed using an individually ventilated caging system under a 12 h light, 12 h dark cycle, with free access to food and water. All surgical interventions and postoperative animal care were carried out in accordance with the guidelines for the care and use of laboratory animals (eighth edition, National Institutes of Health). The experimental protocol was reviewed and approved by the Experimental Animal Ethics Committee of Xuzhou Medical University (Approval No. EA20180302).

### Statistical analysis

The differences between the nontumor group and the glioma subgroups were evaluated using Kruskal-Wallis and Mann-Whitney U tests. Correlations were analyzed by the Pearson’s correlation test. The *in vitro* experiments were repeated at least 3 times, the data are expressed as the mean ± S.E.M., and comparisons between 2 groups were performed using Student’s *t-*test. Animal survival was evaluated using the Kaplan-Meier method, which was followed by the log-rank (Mantel-Cox) test. Statistical analyses were performed using SPSS statistical software for Windows, version 13.0 (SPSS, Chicago, IL, USA). Tests were 2-tailed, and *P* < 0.05 was considered statistically significant.

## Results

### Clinical importance of BYSL in glioma patients

**Figure 1:**
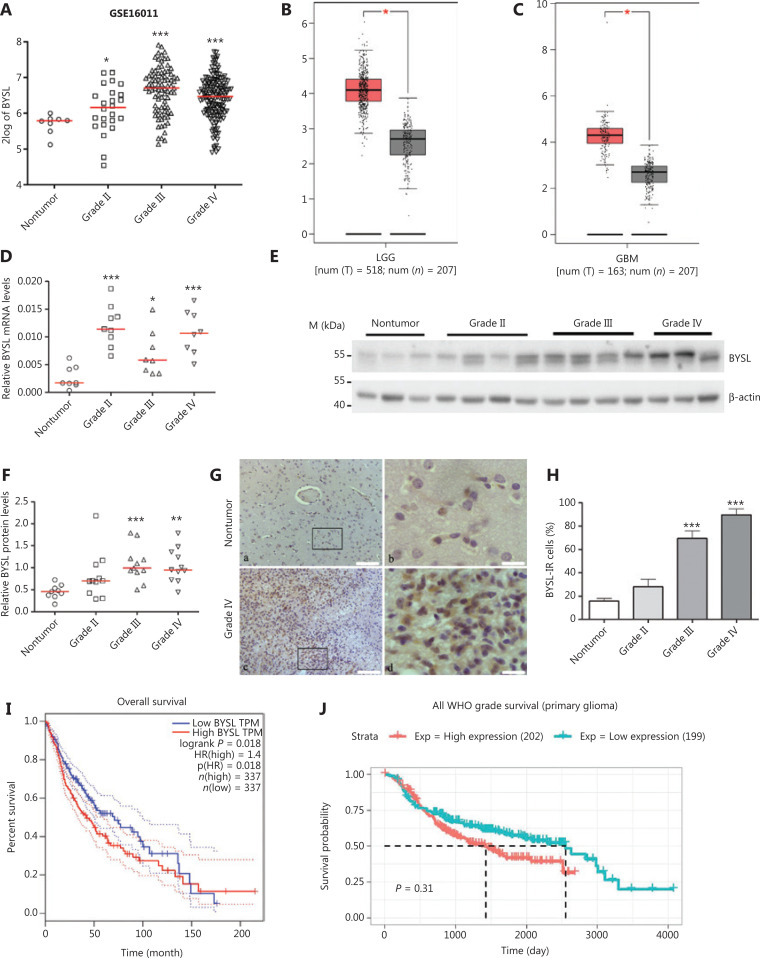
The changes in BYSL expression in glioma tissues and association of BYSL levels with patient survival. (A) Expression analysis of the GSE16011 dataset revealed a significant increase in the BYSL mRNA levels in grade II, grade III, and grade IV glioma tissues compared to those in nontumor tissues. (B, C) Expression analysis with the GEPIA showed that BYSL expression was significantly higher in low grade gliomas and in glioblastomas than those in their respective controls. T: tumor; N: normal. (D) The qRT-PCR analysis showed the upregulation of the BYSL mRNA levels in grade II, grade III, and grade IV glioma tissues compared to those in nontumor tissues. (E, F) Western blot analysis showed an increase in BYSL protein levels in grade II, grade III, and grade IV glioma tissues compared to those in nontumor tissues. (G, H) Immunohistochemistry showed that BYSL immunoreactive (BYSL-IR) signals were distributed in both the cytoplasm and nucleus of tumor cells (G). Scale bars: 100 μm for a and c; 20 μm for b and d. Quantitative analysis of the cell numbers showed increased percentages of BYSL-IR cells in grade II (*n* = 4), grade III (*n* = 4), and grade IV (*n* = 5) glioma tissues relative to those in nontumor tissues (*n* = 5) (H). (I, J) Survival analysis showed that high BYSL levels were associated with poor prognoses in both TCGA (I) and CGGA (J) glioma datasets. M, molecular marker (*P < 0.05; **P < 0.01; ***P < 0.001).

We first analyzed *BYSL* gene expression in the GSE16011 dataset downloaded from the R2 genomics analysis and visualization platform. The BYSL mRNA levels showed a significant increase in grade II (*n* = 24, *P* = 0.043), grade III (*n* = 85, *P* < 0.001) and grade IV (*n* = 159, *P* < 0.001) in glioma tissues when compared to those in nontumor tissues (**Figure 1A**). Expression analyses in the GEPIA Web server showed that the BYSL mRNA levels were higher in LGG and GBM tissues than they were in their respective normal brain tissues (both *P* < 0.05, **Figure 1B, 1C**). Using our cohorts, i.e., 25 glioma tissues (grade II, *n* = 9; grade III, *n* = 8; and grade IV, *n* = 8) and 8 nontumor brain tissues, qRT-PCR assays showed increased BYSL mRNA levels in grade II (*P* < 0.001), grade III (*P* = 0.028), and grade IV (*P* < 0.001) glioma tissues (**Figure 1D**). To determine whether the BYSL mRNA expression profiles reflected protein levels, we next measured the BYSL protein in various grades of glioma tissues. Western blot analysis showed that BYSL protein levels were increased in grade II (*n* = 11, *P* = 0.068), grade III (*n* = 11, *P* < 0.001), and grade IV (*n* = 11, *P* = 0.001) glioma tissues compared to those in nontumor brain tissues (*n* = 9) (**Figure 1E, 1F**). Immunohistochemistry showed that BYSL immunoreactivity was located in both the cytoplasm and nuclei (**Figure 1G**) and that the percentage of BYSL-IR cells was significantly increased in grade III (*P* < 0.001) and grade IV (*P* < 0.001) glioma tissues (**Figure 1H**). In addition, higher BYSL levels were associated with shorter survival times in the TCGA (*n* = 674, *P* = 0.018) and CGGA (*n* = 401, *P* = 0.31) glioma datasets (**Figure 1I, 1J**). These clinical findings suggested the potential involvement of BYSL in the progression of human gliomas.

### Efficiency of BYSL downregulation and overexpression in glioma cells

**Figure 2:**
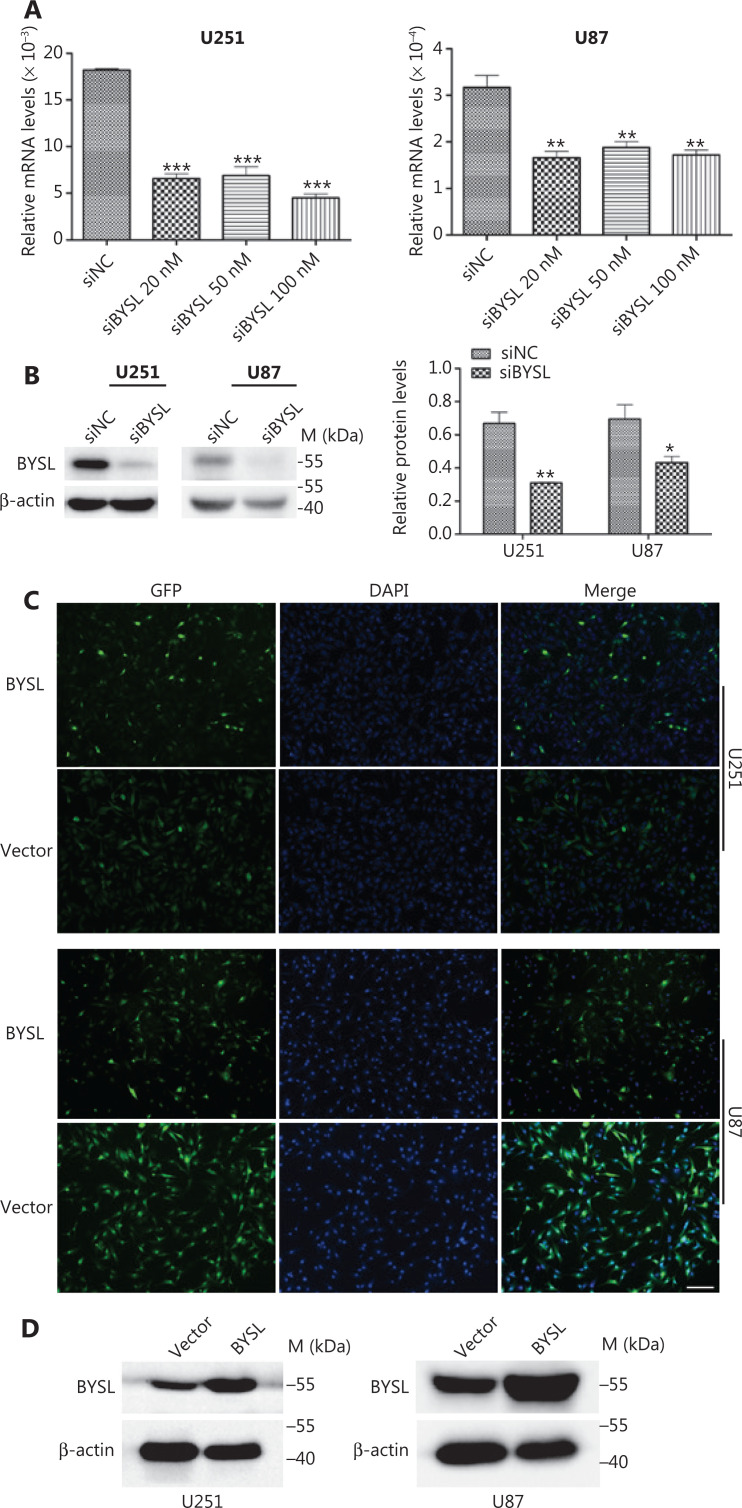
The efficiency of BYSL downregulation and overexpression in glioma cells. (A) The qRT-PCR revealed that different concentrations of BYSL siRNAs significantly downregulated the BYSL levels in U251 and U87 glioma cells. (B) Western blot analysis showed that 100 nM siRNA significantly decreased the BYSL protein levels in U251 and U87 glioma cells. (C) By combining the green fluorescent protein and 4,6-diamidino- 2-phenylindole staining, 90% of the cells were confirmed to be infected by lentiviruses produced from the vector and BYSL plasmids. Scale bar: 200 μm. (D) Western blot analysis showed that BYSL was abundantly expressed in U251 and U87 cells. M, molecular marker (*P < 0.05; **P < 0.01; ***P < 0.001).

Different concentrations of siRNAs were used to transfect glioma cells, and qRT-PCR assays showed that 20 nM, 50 nM, and 100 nM siRNA significantly reduced the BYSL mRNA levels in U251 (all, *P* < 0.001) and U87 (all, *P* < 0.001) cells (**Figure 2A**). Western blot analysis further confirmed the efficiency of downregulation of BYSL at the protein level by 100 nM BYSL siRNA in U251 (*P* = 0.007) and U87 (*P* = 0.048) cells (**Figure 2B**). We established stable glioma cell lines overexpressing BYSL by lentivirus infection of U87 and U251 cells. The fluorescence microscope showed that 90% of the lentivirus-infected cells exhibited GFP fluorescence (**Figure 2C**). Western blot analysis further confirmed that the BYSL protein was abundantly overexpressed in the U251 and U87 cells (**Figure 2D**).

### Effects of BYSL downregulation or overexpression on glioma cell proliferation

**Figure 3:**
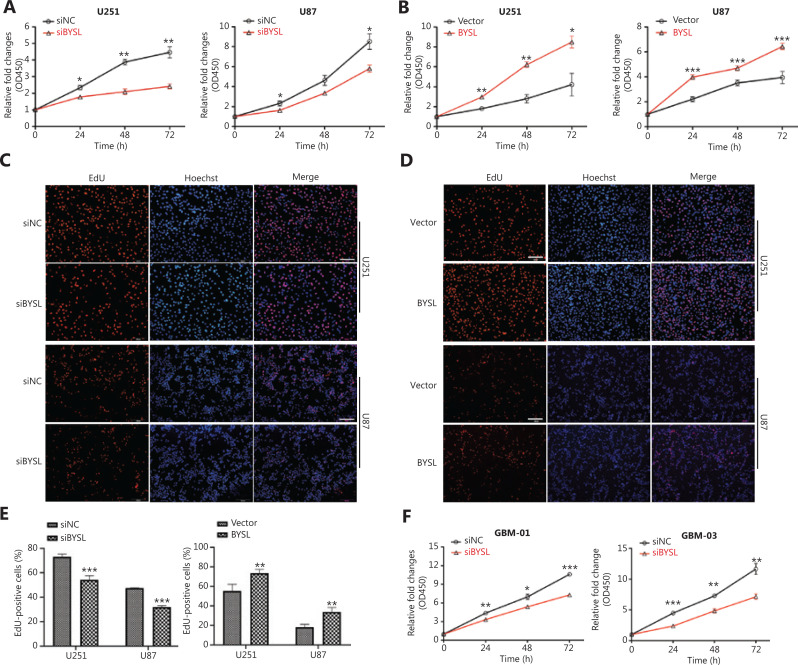
The effects of BYSL downregulation or overexpression on glioma cell growth. (A, B) CCK-8 assays were used to measure the cell viability at 24, 48, and 72 h following BYSL downregulation (A) or overexpression (B) in U251 and U87 cells. (C–E) The 5-ethynyl-20-deoxyuridine (EdU) assays were performed to evaluate the cell proliferation in U251 and U87 cells. Representative images of BYSL downregulation and overexpression are shown in C and D, respectively. Scale bars: 200 μm. Quantitative analyses of the EdU-positive cells with BYSL downregulation and overexpression are shown in E. (F) CCK-8 assays were used to measure the cell viability in primary glioma cells at 24, 48, and 72 h following BYSL downregulation (*P < 0.05; **P < 0.01; ***P < 0.001).

The CCK-8 assay showed that silencing BYSL caused a significant decrease in the cell viability of glioma cells at 24 h (U251: *P* = 0.024; U87: *P* = 0.039), 48 h (U251: *P* = 0.002; U87: *P* = 0.051), and 72 h (U251: *P* = 0.005; U87: *P* = 0.019) (**Figure 3A**), and the cell viabilities of the U251 and U87 cells in the BYSL group were much higher than those in the vector group at 24, 48, and 72 h (U251: *P* = 0.003 at 24 and 48 h; *P* = 0.029 at 72 h; U87: all, *P* < 0.001) (**Figure 3B**). The EdU assay showed a significant inhibitory effect of the downregulation of BYSL in U251 and U87 cells on the percentage of EdU-positive cells (all, *P* < 0.001; **Figure 3C, 3E**), while overexpressing BYSL in U251 and U87 cells resulted in a significant increase in the percentage of EdU-positive cells (U251: *P* = 0.003; U87: *P* = 0.007) (**Figure 3D, 3E**). In addition, knockdown of BYSL significantly decreased the cell viability in primary glioma cells at 24 h (GBM-01: *P* = 0.002; GBM-03: *P* < 0.001), 48 h (GBM-01: *P* = 0.011; GBM-03: *P* = 0.005), and 72 h (GBM-01: *P* < 0.001; GBM-03: *P* = 0.004) (**Figure 3F**). These data suggested that BYSL promoted glioma cell proliferation.

### The effects of BYSL downregulation or overexpression on cell cycle progression in glioma cells

**Figure 4:**
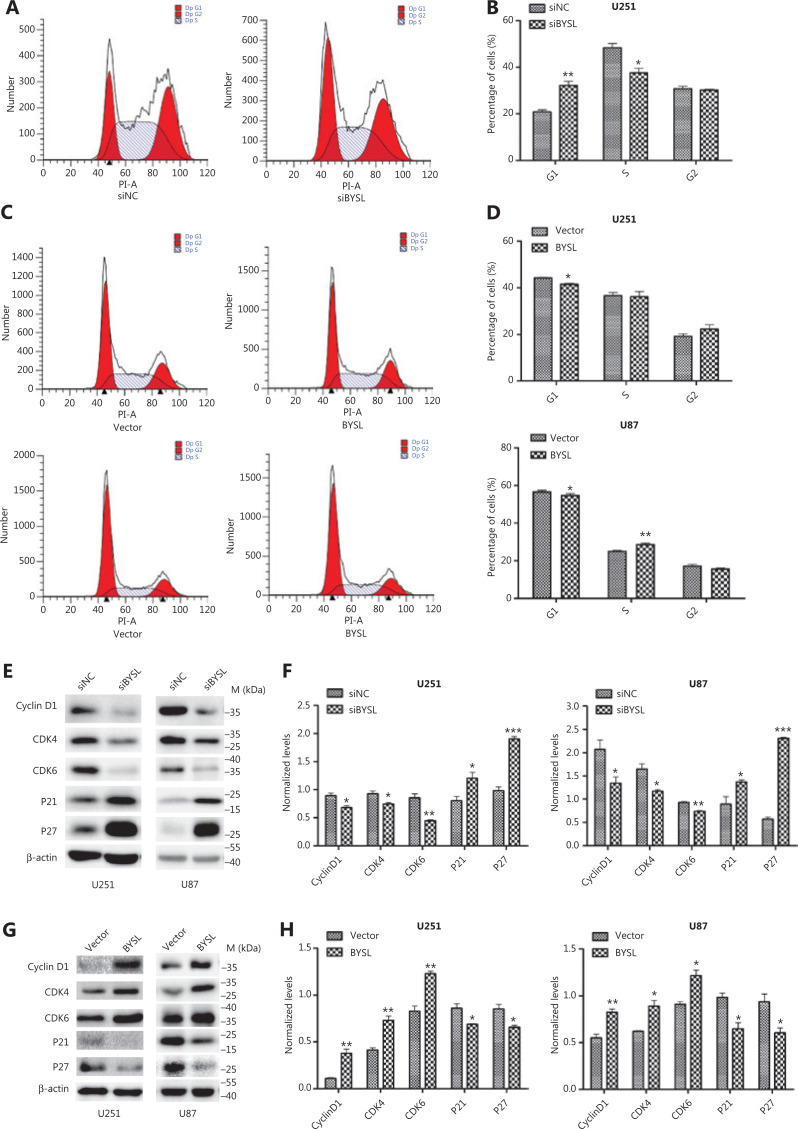
The effects of BYSL downregulation or overexpression on cell cycle progression in glioma cells. (A–D) Flow cytometry was used to analyze the cell cycle progression in U251 and U87 cells. Representative histograms of BYSL downregulation and overexpression are shown in A and C, respectively. Quantitative analyses of the percentage of cells in G1, S, and G2 phases are shown in B (BYSL downregulation) and D (BYSL overexpression). (E–H) Western blot analysis was used to measure the changes in the cell cycle-related proteins in U251 and U87 cells. Representative blot images of BYSL downregulation and overexpression are shown in E and G, respectively. Graphs quantifying the blot images of BYSL downregulation and overexpression are shown in F and H, respectively. M, molecular marker (*P < 0.05; **P < 0.01; ***P < 0.001).

Flow cytometry showed that introducing BYSL siRNAs into U251 cells increased the percentage of cells in G1 phase (*P* = 0.004) and decreased the percentage of cells in S phase (*P* = 0.017) without affecting the percentage of cells in G2 phase (**Figure 4A, 4B**). In comparison, overexpressing BYSL decreased the percentage of cells in G1 phase (U251: *P* = 0.010; U87: *P* = 0.013), and increased the percentage of U251 cells in G2 phase (*P* = 0.096) and the percentage of U87 cells in S phase (*P* = 0.004) (**Figure 4C, 4D**). These findings indicated that BYSL promoted cell cycle progression in glioma cells.

Silencing of BYSL significantly decreased the protein levels of cyclin D1 (*P* = 0.019) and cyclin-dependent kinases (CDKs), such as CDK4 (*P* = 0.026) and CDK6 (*P* = 0.005), and significantly increased CDK inhibitors (CDKIs), such as P21 (*P* = 0.037) and P27 (*P* < 0.001), in U251 cells (**Figure 4E, 4F**). We observed similar changes in U87 cells, i.e., significant decreases in cyclin D1 (*P* = 0.041), CDK4 (*P* = 0.017), and CDK6 (*P* = 0.001) levels and significant increases in P21 (*P* = 0.046) and P27 (*P* < 0.001) levels (**Figure 4E, 4F**). In contrast, the overexpression of BYSL led to significant increases in cyclin D1 (*P* = 0.004), CDK4 (*P* = 0.004), and CDK6 (*P* = 0.003) protein levels and significant decreases in P21 (*P* = 0.032) and P27 (*P* = 0.024) protein levels in U251 cells (**Figure 4G, 4H**). We observed similar changes in U87 cells, i.e., significant increases in cyclin D1 (*P* = 0.006), CDK4 (*P* = 0.013), and CDK6 (*P* = 0.010) protein levels and significant decreases in the P21 (*P* = 0.013) and P27 (*P* = 0.027) (**Figure 4G, 4H**). These data suggested that BYSL promoted the G1-to-S transition in glioma cells.

### Downregulation of BYSL induces apoptosis in glioma cells

**Figure 5:**
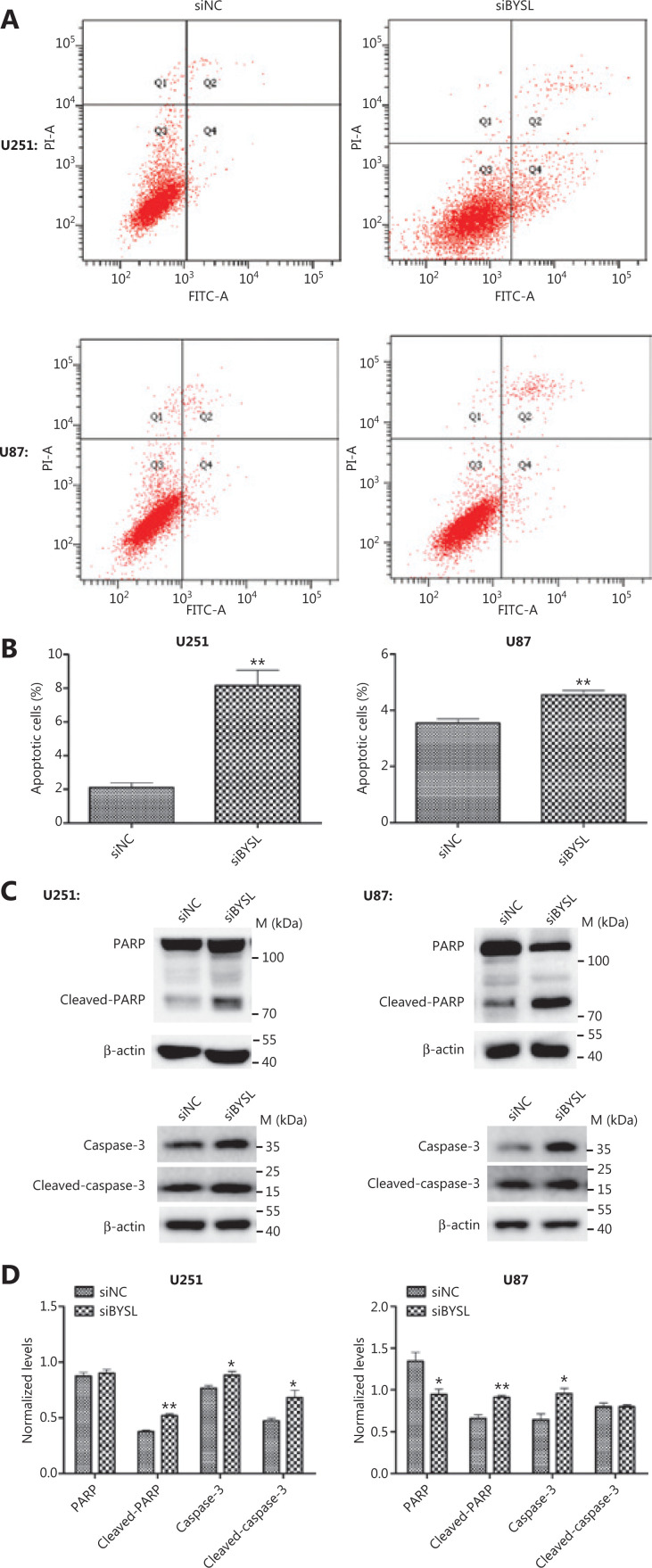
The effects of BYSL downregulation on glioma cell apoptosis. (A, B) Flow cytometry was used to analyze apoptosis in U251 and U87 cells. Representative histograms are shown in A. Quantitative analyses of the percentage of apoptotic cells are shown in B. (C, D) Western blot analyses of caspase-3 and PARP levels were used to evaluate apoptosis in U251 and U87 cells. Representative blot images are shown in C. Graphs quantifying the blot images are shown in D. M, molecular marker (*P < 0.05; **P < 0.01).

Flow cytometry apoptosis assays revealed that silencing BYSL resulted in a significant increase in the percentage of apoptotic cells (U251: *P* = 0.003; U87: *P* = 0.001) (**Figure 5A, 5B**). Because the cleavage of PARP by caspase-3 is known as a universal hallmark of apoptotic cell death^24^, we next analyzed the PARP and caspase-3 levels in glioma cells by Western blot analysis. Silencing BYSL significantly increased the cleaved PARP (*P* = 0.001), caspase-3 (*P* = 0.044), and cleaved caspase-3 (*P* = 0.042) levels in U251 cells (**Figure 5C, 5D**). In U87 cells, the downregulation of BYSL led to a significant decrease in the PARP (*P* = 0.034) levels and significant increases in the cleaved PARP (*P* = 0.007) and caspase-3 (*P* = 0.025) levels (**Figure 5C, 5D**). These results suggested that silencing BYSL induced apoptosis in glioma cells.

### BYSL forms a complex with RIOK2 and mTOR

**Figure 6:**
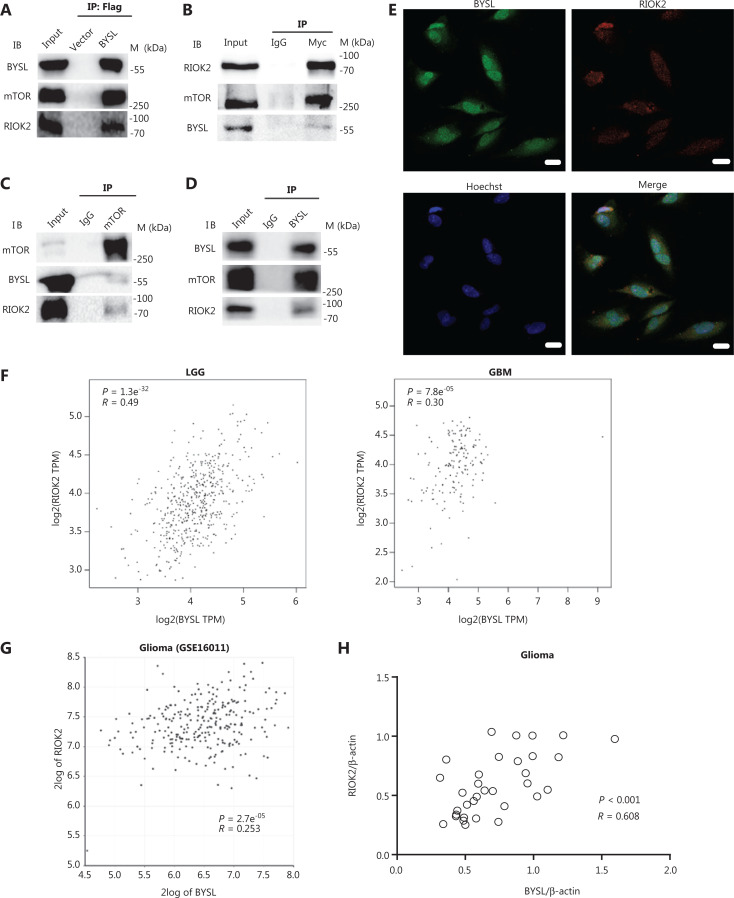
The association of BYSL with RIOK2 and mTOR. (A) FLAG-BYSL plasmid was transfected into HEK293T cells. Immunoprecipitation (IP) with FLAG magnetic beads showed that mTOR and RIOK2 were associated with BYSL. (B) The Myc-RIOK2 plasmid was transfected into HEK293T cells. IP with the anti-Myc antibody showed that mTOR and BYSL were associated with RIOK2. (C) FLAG-BYSL plasmid was transfected into HEK293T cells. IP with the anti-mTOR antibody showed that RIOK2 and BYSL were associated with mTOR. (D) IP with the anti-BYSL antibody showed the association of BYSL with RIOK2 and mTOR in U251 cells. (E) Double labeling immunofluorescence was performed in U251 glioma cells. BYSL partially co-localized with RIOK2 in both the cytoplasm and nuclei. Scale bar: 20 μm. (F, G) Correlation analysis between BYSL and RIOK2 at the gene level in the LGG, GBM, and GSE16011 datasets. (H) Correlation analysis between BYSL and RIOK2 at the protein level in our glioma samples. The correlation efficiency and *P*-values are indicated in the figures. M, molecular marker.

To elucidate the molecular mechanism by which BYSL promoted glioma cell growth and survival, we analyzed the STRING and IntAct databases. It was suggested that BYSL may interact with an atypical kinase, RIOK2. We next performed a co-immunoprecipitation assay in HEK293T cells and found that exogenous BYSL formed a complex with RIOK2 and mTOR (**Figure 6A**) and that exogenous RIOK2 also formed a complex with BYSL and mTOR (**Figure 6B**). In addition, endogenous mTOR co-immunoprecipitated with RIOK2 and BYSL in BYSL-overexpressing cells (**Figure 6C**) and endogenous BYSL co-immunoprecipitated with RIOK2 and mTOR in U251 glioma cells (**Figure 6D**). Double labeling immunofluorescence showed that both BYSL and RIOK2 were localized to the cytoplasm and nuclei, and their immunoreactivities partially overlapped in U251 cells (**Figure 6E**). Furthermore, we found a significantly positive correlation between *BYSL* and *RIOK2* gene expressions in LGG (*R* = 0.49, *P* = 1.3e^-32^), GBM (*R* = 0.30, *P* = 7.8e^-05^), and various grades of glioma tissues (*R* = 0.253, *P* = 2.7e^-05^) by analyzing public datasets (**Figure 6F, 6G**). Western blot analysis of our cohorts also showed a significantly positive correlation between BYSL and RIOK2 expressions in glioma tissues (*n* = 33, *R* = 0.608, *P* < 0.001) but not in nontumor brain tissues (*n* = 9, *R* = 0.377, *P* = 0.317) (**Figure 6H**). These data suggested that BYSL formed a complex with RIOK2 and mTOR in glioma cells.

### BYSL and RIOK2 positively regulate AKT/mTOR signaling in glioma cells

RIOK2 was reported to mediate cell proliferation and survival through AKT signaling in GBM cells^25^. We next established lentivirus-mediated glioma cell lines that exhibited stable overexpression/downregulation of RIOK2. The infection efficiency was verified in both U251 and U87 cells (**Supplementary Figures S1, S2**). ShRIOK2-2 was used for the following experiments. We confirmed previous findings^25^ in these cell lines, i.e., the phosphorylation of AKT (308), AKT (473), and PARS40 was increased when RIOK2 was overexpressed, and the phosphorylation of these molecules was decreased when RIOK2 was downregulated (**Supplementary Figure S3**). Moreover, the overexpression of RIOK2 increased the total levels of mTOR and the phosphorylation of mTOR, P70S6K, and S6, while the silencing of RIOK2 decreased the phosphorylation of these molecules in U251 and U87 cells (**Supplementary Figure S3**).

**Figure 7:**
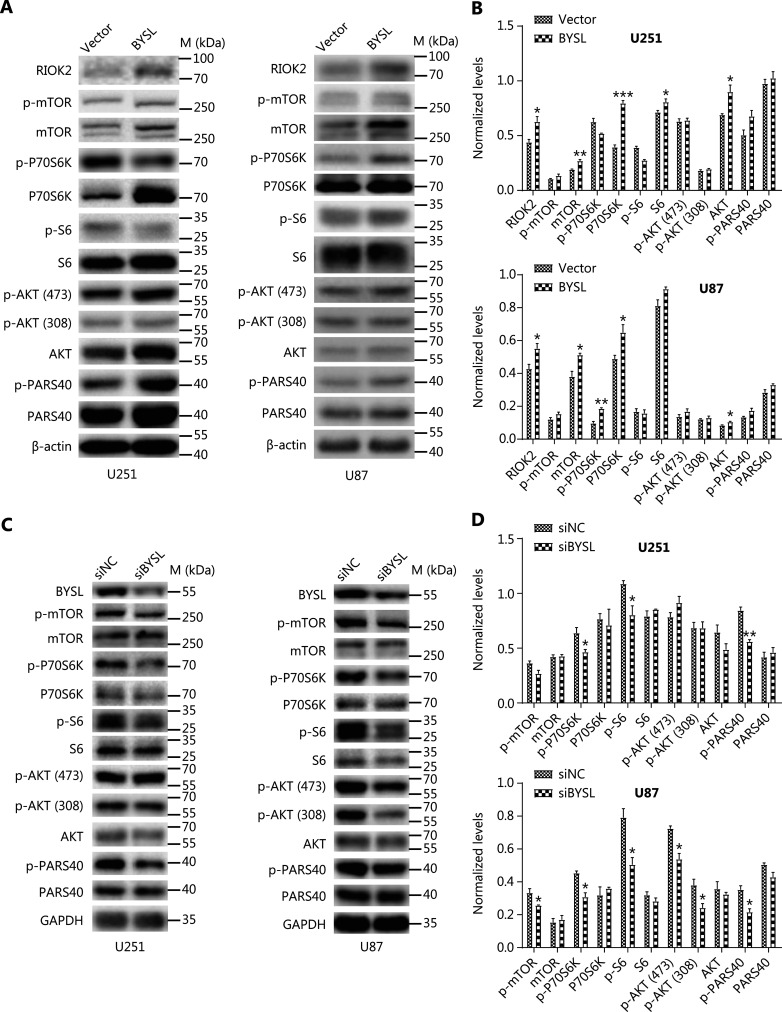
The effects of BYSL overexpression or downregulation on the AKT and mTOR signaling pathways in glioma cells. Western blot analysis was used to measure the levels of the total and phosphorylated AKT/mTOR signaling molecules in U251 and U87 cells. Representative blot images of BYSL overexpression and downregulation are shown in A and C, respectively. Graphs quantifying the blot images of BYSL overexpression and downregulation are shown in B and D, respectively. M, molecular marker (*P < 0.05; **P < 0.01; ***P < 0.001).

Because BYSL, RIOK2, and mTOR existed in the same complex (**Figure 6**), we next identified the role of BYSL in regulating RIOK2, AKT, and mTOR signaling in glioma cells. RIOK2 (*P* = 0.026), mTOR (*P* = 0.006), P70S6K (*P* < 0.001), S6 (*P* = 0.049), and AKT (*P* = 0.031) levels were significantly increased in BYSL-overexpressing U251 cells, and these findings, i.e., increased RIOK2 (*P* = 0.041), mTOR (*P* = 0.018), P70S6K (*P* = 0.041), p-P70S6K (*P* = 0.003), S6 (*P* = 0.057), AKT (*P* = 0.017), and PARS40 (*P* = 0.066) levels, were further verified in the BYSL-overexpressing U87 cells (**Figure 7A, 7B**). However, the overexpression of BYSL led to insignificant changes in the phosphorylation of mTOR, AKT (308 or 473), and PARS40 in either the U251 or U87 cells, and even led to an insignificant reduction in the phosphorylation of P70S6K and S6 in U251 cells (**Figure 7A, 7B**). Moreover, transfection of FLAG-BYSL plasmid into U251 cells significantly increased the total levels of mTOR (*P* = 0.008) and AKT (*P* < 0.001), without affecting the phosphorylation of mTOR or AKT (308 or 473) (**Supplementary Figure S4**). These results indicated that either stable or transient overexpression of BYSL elevated the protein levels of the RIOK2/AKT/mTOR signaling molecules in glioma cells.

Silencing of BYSL significantly decreased the phosphorylation of mTOR (*P* = 0.058), P70S6K (*P* = 0.036), S6 (*P* = 0.033), and PARS40 (*P* = 0.002) in U251 cells, and these results, i.e., reduced phosphorylation of mTOR (*P* = 0.039), P70S6K (*P* = 0.037), S6 (*P* = 0.014), PARS40 (*P* = 0.012), and AKT at both 308 (*P* = 0.040) and 473 (*P* = 0.040) sites, were also confirmed in U87 cells (**Figure 7C, 7D**). These data suggested that silencing BYSL downregulated the activity of the AKT/mTOR signaling pathway in glioma cells.

### Downregulation of BYSL or RIOK2 inhibits glioma growth in an orthotopic xenograft model

**Figure 8:**
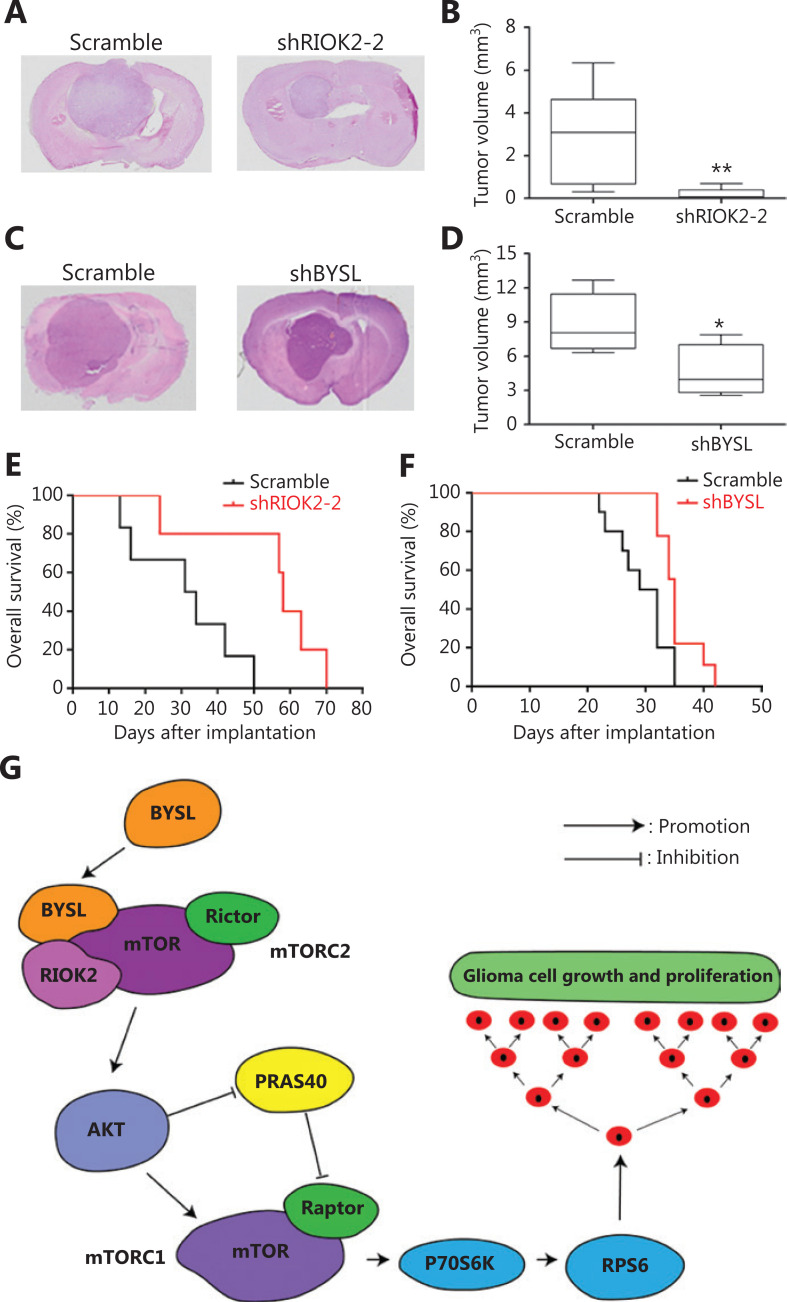
The effects of BYSL or RIOK2 downregulation on glioma growth *in vivo*. An orthotopic xenograft model was established in nude mice. (A–D) Hematoxylin and eosin (H&E) staining was performed with whole brain sections from tumor-bearing mice. Representative H&E-stained images for the shRIOK2-2 and shBYSL groups are shown in A and C, respectively (100×). Graphs showed that silencing RIOK2 (B) or BYSL (D) significantly decreased the tumor volume. (E, F) Survival analysis showed that silencing RIOK2 or BYSL significantly prolonged the overall survival time of tumor-bearing mice. (G) A working model. BYSL and RIOK2 exist in the same complex with mTORC2, which is in turn connected to the AKT-mTORC1-P70S6K signaling, and thus increases ribosomal protein (e.g., S6) synthesis, leading to glioma cell growth and proliferation (*P < 0.05; **P < 0.01).

An intracranial xenograft model was established in nude mice. The downregulation of RIOK2 significantly inhibited the growth of tumors (*P* = 0.009, **Figure 8A, 8B**) and prolonged overall animal survival (*P* = 0.017, **Figure 8E**). Similarly, silencing BYSL led to decreased tumor volume (*P* = 0.030, **Figure 8C, 8D**) and longer survival time (*P* = 0.014, **Figure 8F**). These results suggested that silencing BYSL or RIOK2 inhibited glioma growth *in vivo*.

## Discussion

In the present study, we demonstrated that BYSL mRNA and protein levels were upregulated in glioma tissues, especially in high grade gliomas. The downregulation of BYSL inhibited proliferation, impeded cell cycle progression, and induced apoptosis in glioma cells, while the overexpression of BYSL led to the opposite effects. In addition, experiments with a glioma orthotopic xenograft model showed that silencing BYSL or RIOK2 decreased the growth of tumors and prolonged the survival times of tumor-bearing mice. Mechanistically, BYSL formed a complex with RIOK2 and mTOR, the overexpression of BYSL increased the expression of RIOK2, AKT, PARS40, mTOR, P70S6K, and S6, while silencing of BYSL decreased the activity of the AKT/mTOR pathway (**Figure 8G**). These findings suggested that BYSL functioned as a critical component of the RIOK2-mTOR complex and that interfering with BYSL may provide a promising strategy for glioma treatments.

BYSL expression was previously found to be elevated in lung^26^, liver^6^, prostate^8^, and ovarian cancers^7^. Here, we showed a significant increase in BYSL expression in various grades of glioma tissues, especially in high grade gliomas, by analyzing public datasets and our cohorts. More importantly, high levels of BYSL mRNA were associated with poor prognoses, as observed in the glioma datasets from both TCGA and CGGA. It should be noted that BYSL is also upregulated in reactive astrocytes in response to brain injury and/or inflammation^10,11^. Therefore, BYSL may be a useful biomarker for astrocyte proliferation, but it cannot distinguish between glioma and gliosis.

*BYSL* is a gene that is highly evolutionarily conserved from yeast to humans^3,4^; it encodes the Bystin protein in humans. BYSL is involved in 18S pre-rRNA processing and 40S ribosomal subunit biogenesis in both yeast and humans^4,27^. The relocalization and reactivation of the pre-rRNA processing machinery are critical for nucleolar assembly. Wang et al.^6^ proposed that knockdown of BYSL may impair nucleogenesis, leading to inhibition of HCC cell growth. The dynamic assembly of nucleoli is an important event in the exit of mitosis in eukaryotic cells. Our data showed that silencing BYSL caused cell cycle arrest at G1 phase in glioma cells, which was further verified by the downregulation of cyclin D1 and CDKs (CDK4 and CDK6) and the upregulation of CDKIs (P21 and P27). Therefore, knockdown of BYSL may impair pre-rRNA processing and nucleolar assembly, thereby impeding mitotic progression and proliferation in glioma cells.

The current study aimed to identify the molecular mechanism by which BYSL promoted glioma cell growth. By analyzing the String and Intact databases, we found that BYSL may interact with an atypical kinase, RIOK2. Our co-immunoprecipitation experiments confirmed this prediction. RIOK2, which is a member of the RIO family, plays a key role in the synthesis of the 40S ribosomal subunits by promoting the maturation of 18S rRNA^28^. The functional similarity of BYSL and RIOK2 in ribosomal biosynthesis provides a rationale for their existence in the same complex. In addition, BYSL is co-expressed with RIOK2 in glioma cells and is positively correlated with RIOK2 at both the gene and protein levels in glioma tissues. Because a previous study demonstrated that the overexpression of RIOK2 promoted proliferation and tumorigenesis, whereas the silencing of RIOK2 inhibited the proliferation and survival of glioma cells and prolonged the survival time of glioma-bearing mice^25^, these findings suggested that BYSL may function by regulating RIOK2 to promote glioma cell growth.

RIOK2 was reported to be specifically associated with mTORC2, because it co-immunoprecipitated with Rictor (a characteristic component of mTORC2), instead of Raptor (a characteristic component of mTORC1)^25^. Here, we also confirmed the interaction between RIOK2 and mTOR. Moreover, our co-immunoprecipitation analyses showed that BYSL interacted with RIOK2 and mTOR in both HEK293T and U251 cells. Therefore, both BYSL and RIOK2 existed in the mTORC2 complex. It has been found that the overexpression of RIOK2 upregulates mTORC2-AKT activity, whereas the loss of RIOK2 disrupts this signaling^25^. Here, we extended the promoting role of RIOK2 to downstream of the mTORC2-AKT signaling pathway, i.e., the mTOR-P70S6K-S6 pathway. Thus, it is expected that manipulating BYSL would affect both the AKT and mTOR signaling pathways. Indeed, the overexpression of BYSL caused a significant increase in the protein levels of RIOK2, AKT, PARS40, mTOR, P70S6K, and S6 in glioma cells. However, it remains to be determined whether the accumulation of the AKT/mTOR signaling molecules is due to increased protein synthesis or decreased protein degradation, thereby increasing protein stability.

The mTORC1-P70S6K-S6 signaling plays a significant role in accelerating ribosome synthesis^29^. RIOK2 participates in 18S rRNA processing and 40S ribosomal biosynthesis^28^. Therefore, we propose that the BYSL-mediated RIOK2/mTOR/AKT signaling network might be responsible for the key role of BYSL in multiple biological processes, such as pre-rRNA processing, nucleolar assembly, and ribosome synthesis, all of which are necessary for the unregulated proliferation of gliomas. This proposal requires additional investigation.

We found that silencing BYSL decreased the phosphorylation of AKT, PARS40, mTOR, P70S6K, and S6 without affecting the protein levels of these signaling molecules. This finding provided deep insights into the treatment of glioma through the targeting of BYSL. Most primary GBMs harbor EGFR amplification and/or PTEN deletion(s)^30^, resulting in abnormal activation of the PI3K/AKT pathway. Drugs that target multiple aspects of this pathway in glioma have been used in the clinic or in ongoing clinical trials (https://clinicaltrials.gov/ct2/home). Nevertheless, the development of an antitumor drug that targets an upstream driver of the AKT/mTOR pathway (e.g., BYSL or RIOK2) will reduce the drug toxicity caused by targeting a combination of different molecules in the pathway, and may possibly achieve precise treatment for glioma patients with EGFR amplification and/or PTEN deficiency.

A few limitations of this study should be mentioned. We demonstrated that overexpression of BYSL elevated the RIOK2 and mTOR protein levels and that they existed in the same complex in glioma cells, but we did not show whether their interactions were enhanced in glioma cells/tissues that highly expressed BYSL. Although we found that silencing BYSL significantly inhibited cell growth in the primary GBM cell lines, further investigations in patient-derived xenograft models are needed to confirm this effect *in vivo*.

## Conclusions

High expression of BYSL in glioma tissues promoted tumor cell growth and survival. BYSL existed in the same complex as RIOK2 and mTOR. Both BYSL and RIOK2 positively regulated AKT/mTOR signaling, which might be responsible for the promoting effects of BYSL on human glioma growth (**Figure 8G**). These results suggested that BYSL was a potential oncogene in glioma and that it may serve as a therapeutic target for this disease.

## Grant support

This work was supported by the National Natural Science Foundation of China (Grant Nos. 82002632 and 82072763) and the Key Research & Development Plan of Jiangsu Province (Grant No. BE2017636) and Xuzhou City (Grant No. KC20076). S. Gao was supported by the Six Talents Peak (Grant No. 2019-SWYY-092), the Medical Youth Talent (Grant No. QNRC2016787), and the Qing Lan projects of Jiangsu Province.
