# Supplementary material for: BYSL contributes to tumor growth by cooperating with the mTORC2 complex in gliomas
Source: Cancer Biol Med. 2021 Feb 15;18(1):88–104. doi: 10.20892/j.issn.2095-3941.2020.0096 (PMC7877178; doi:10.20892/j.issn.2095-3941.2020.0096)
Supplement: Supplementary file 1 [file cbm-18-088-s001.pdf]

Supplementary materials

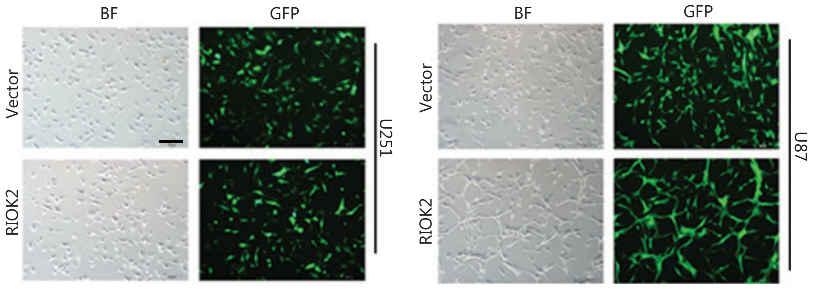

Figure S1 The efficiency of RIOK2 overexpression in glioma cells. The representative bright field and fluorescent field images of the vector and RIOK2 groups in U251 and U87 cells. Scale bar: 200 μm.

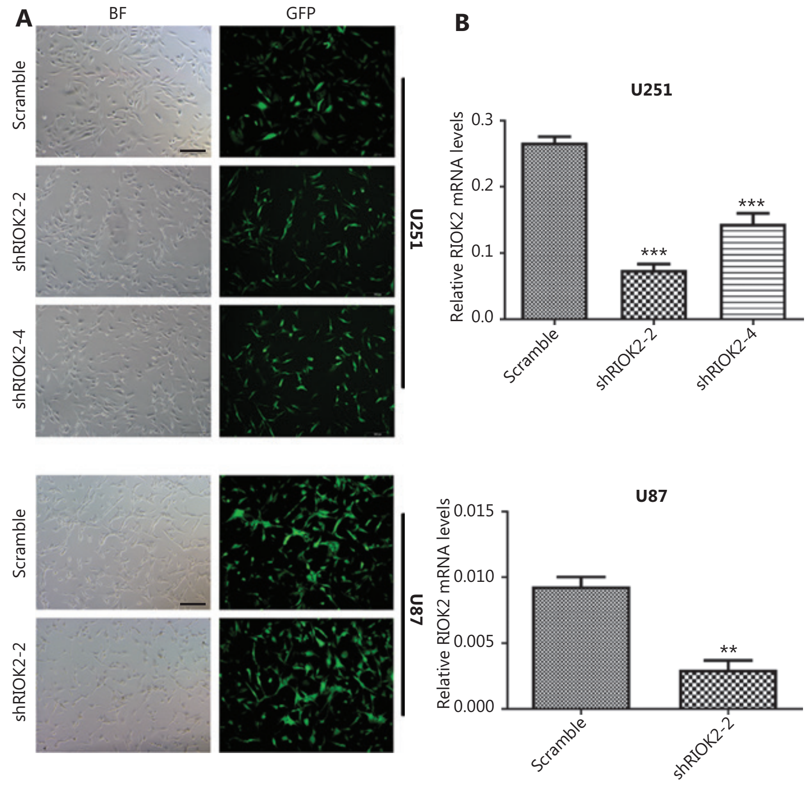

Figure S2 The efficiency of RIOK2 downregulation in glioma cells. (A) The representative bright field and fluorescent field images of the scramble and shRIOK2 groups in U251 and U87 cells. Scale bar: 200 μm. (B) The qRT-PCR revealed that silencing of RIOK2 significantly down-regulated the RIOK2 mRNA levels (***P* < 0.01; ****P* < 0.001).

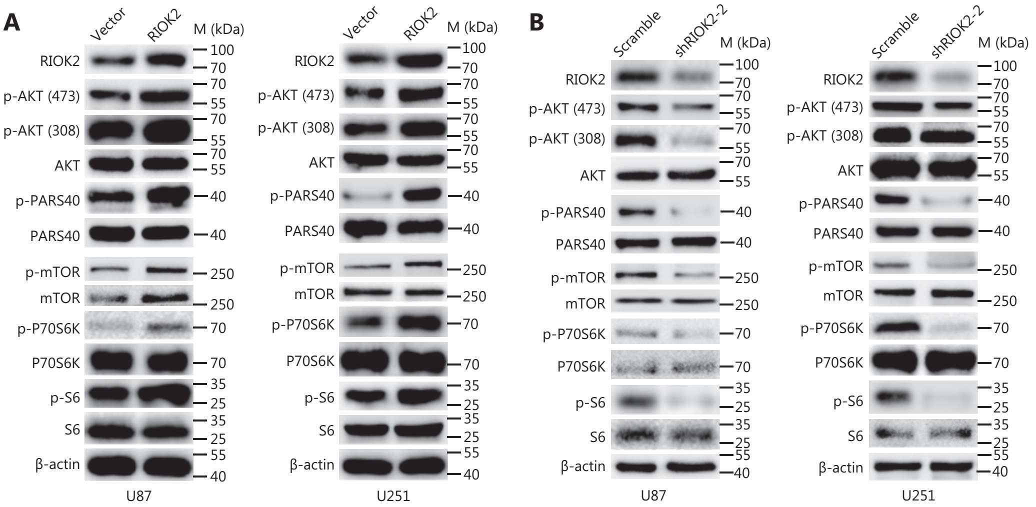

Figure S3 The effects of RIOK2 knockdown or overexpression on the AKT and mTOR signaling in glioma cells. Western blot analyses were performed in U251 and U87 cells. (A) Overexpression of RIOK2 led to an increase in the p-AKT (473 and 308), p-PRAS40, p-mTOR, mTOR, p-P70S6K, and p-S6 levels. (B) Silencing of RIOK2 caused a decrease in the p-AKT (473 and/or 308), p-PARS40, p-mTOR, p-P70S6K and p-S6 levels. M, molecular marker.

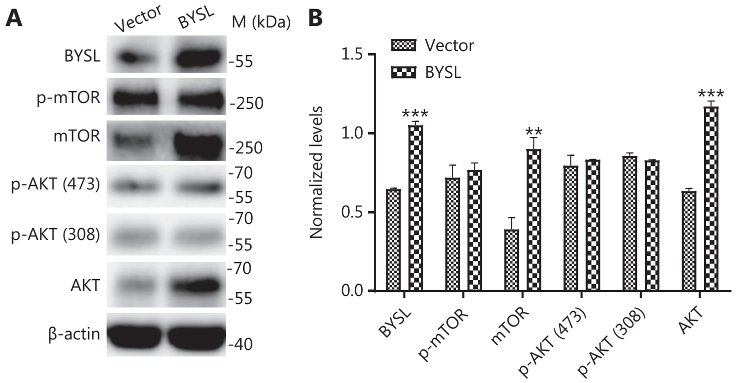

Figure S4 The effects of FLAG-BYSL plasmid transfected into glioma cells on AKT and mTOR signaling. Western blot analysis was performed in U251 cells. Representative blot images of FLAG-BYSL plasmid transfection are shown in A. Graphs quantifying the blot images of FLAG-BYSL plasmid transfection are shown in B. M, molecular marker (***P* < 0.01; ****P* < 0.001).

Table S1 Clinicopathological information for the studied subjects

Case No.	Code No.	Gender	Age (years)	Used for	WHO grade
1	912226	F	54	WB	Nontumor
2	928412	M	48	WB	Nontumor
3	941814	M	20	WB	Nontumor
4	968605	F	69	WB	Nontumor
5	971400	F	30	WB, IHC	Nontumor
6	972078	F	49	WB, IHC, qRT-PCR	Nontumor
7	981488	M	41	WB	Nontumor
8	1004728	M	63	WB, IHC, qRT-PCR	Nontumor
9	1024827	F	46	WB	Nontumor
10	1026751	M	66	IHC	Nontumor
11	1095392	M	32	WB, IHC, qRT-PCR	Nontumor
12	1233247	F	49	qRT-PCR	Nontumor
13	1237928	M	53	qRT-PCR	Nontumor
14	1241213	F	54	qRT-PCR	Nontumor
15	1615150	M	40	qRT-PCR	Nontumor
16	1619563	F	17	qRT-PCR	Nontumor
17	970570	M	52	WB	Grade II
18	1019453	F	43	IHC	Grade II
19	1029589	M	31	WB	Grade II
20	1031768	M	23	IHC	Grade II
21	1049476	M	50	IHC	Grade II
22	1052079	F	47	IHC	Grade II
23	1110685	F	27	WB	Grade II
24	1140811	F	49	WB	Grade II
25	1145933	F	48	WB	Grade II
26	1152968	F	63	WB	Grade II
27	1157139	M	42	WB, qRT-PCR	Grade II
28	1158620	M	43	WB, qRT-PCR	Grade II
29	1161905	M	73	qRT-PCR	Grade II
30	1164493	M	64	WB, qRT-PCR	Grade II
31	1174937	M	27	qRT-PCR	Grade II
32	1190502	M	31	WB, qRT-PCR	Grade II
33	1196273	M	40	WB, qRT-PCR	Grade II
34	1197520	M	40	qRT-PCR	Grade II
35	1245545	M	27	qRT-PCR	Grade II

Table S1 (continued)

Case No.	Code No.	Gender	Age (years)	Used for	WHO grade
36	920498	M	50	WB	Grade III
37	922050	F	19	WB	Grade III
38	926714	M	56	WB	Grade III
39	947804	F	66	WB	Grade III
40	986708	M	47	WB	Grade III
41	999737	M	52	WB, IHC	Grade III
42	1020742	F	35	IHC	Grade III
43	1032540	F	69	IHC	Grade III
44	1081283	F	58	WB	Grade III
45	1117547	M	47	WB	Grade III
46	1145935	M	23	WB	Grade III
47	1164248	F	66	WB, qRT-PCR	Grade III
48	1182956	M	48	IHC	Grade III
49	1191197	M	68	WB, qRT-PCR	Grade III
50	1234213	F	59	qRT-PCR	Grade III
51	1241430	F	19	qRT-PCR	Grade III
52	1246522	M	34	qRT-PCR	Grade III
53	1260826	M	78	qRT-PCR	Grade III
54	1315290	M	39	qRT-PCR	Grade III
55	1413682	F	44	qRT-PCR	Grade III
56	947968	M	62	IHC	Grade IV
57	966838	M	50	IHC	Grade IV
58	1075388	M	58	IHC	Grade IV
59	1077922	M	43	WB	Grade IV
60	1086721	F	64	IHC	Grade IV
61	1088070	F	58	WB, IHC	Grade IV
62	1096129	M	61	WB	Grade IV
63	1119597	F	50	WB	Grade IV
64	1132842	F	62	WB	Grade IV
65	1140776	M	26	WB	Grade IV
66	1141904	M	47	WB	Grade IV
67	1147166	M	58	WB	Grade IV
68	1147279	F	64	WB	Grade IV
69	1164493	M	66	WB	Grade IV
70	1184604	F	34	WB	Grade IV

Table S1 (continued)

Case No.	Code No.	Gender	Age (years)	Used for	WHO grade
71	1204760	F	65	qRT-PCR	Grade IV
72	1204815	M	43	qRT-PCR	Grade IV
73	1223892	M	66	qRT-PCR	Grade IV
74	1242288	M	26	qRT-PCR	Grade IV
75	1252386	F	54	qRT-PCR	Grade IV
76	1271138	M	30	qRT-PCR	Grade IV
77	1516156	M	49	qRT-PCR	Grade IV
78	1550918	M	42	qRT-PCR	Grade IV

Note: F, female; M, male; WB, Western blot; qRT-PCR, quantitative real-time PCR; IHC, immunohistochemistry; WHO, World Health Organization.
